# Supplementary material for: Hepatitis E as a cause of adult hospitalization in Bangladesh: Results from an acute jaundice surveillance study in six tertiary hospitals, 2014-2017
Source: PLoS Negl Trop Dis. 2020 Jan 21;14(1):e0007586. doi: 10.1371/journal.pntd.0007586 (PMC6994197; doi:10.1371/journal.pntd.0007586)
Supplement: S1 Table — (DOCX) [file pntd.0007586.s002.docx]

| Symptom | Is symptom present at any time during current illness?  (Yes/No)  n (%) | How many days ago did patient first experience symptom?  (days)  Median (IQR) | Is symptom continuing on the day of hospital admission?  (Yes/No)  n (%) |
| --- | --- | --- | --- |
| Fever | 1717 (89%) | 15 (8-30) | 794 (41%)) |
| Yellow skin | 1918 (100%) | 12 (7-21) | 1901 (99%) |
| Yellow eyes | 1922 (100%) | 14 (7-25) | 1916 (100%) |
| Dark color urine | 1910 (99%) | 15 (5-30) | 1892 (98%) |
| Nausea | 1602 (83%) | 12 (7-21) | 898 (47%) |
| Vomiting | 1304 (68%) | 8 (5-15) | 398 (21%) |
| Loss of appetite | 1556 (81%) | 15 (9-30) | 1407 (73%) |
| Abdominal pain | 1175 (61%) | 12 (7-28) | 886 (43%) |
| Haematemesis | 74 (4%) | 5 (3-10) | 16 (1%) |
| Diarrhoea | 327 (17%) | 7 (4-15) | 77 (4%) |
| Constipation | 826 (43%) | 10 (7-20) | 492 (26%) |
| Melaena | 367 (19%) | 7 (4-15) | 123 (6%) |
| Headache | 694 (36%) | 10 (7-20) | 433 (23%) |
| Convulsion | 161 (8%) | 5 (2-10) | 43 (2%) |
| Unconsciousness | 152 (8%) | 3 (2-6) | 62 (3%) |
| Drowsiness | 776 (40%) | 15 (7-30) | 700 (36%) |
| Altered mental status | 146 (8%)) | 10 (4-30) | 109 (6%) |
