# Supplementary material for: Hepatitis E as a cause of adult hospitalization in Bangladesh: Results from an acute jaundice surveillance study in six tertiary hospitals, 2014-2017
Source: PLoS Negl Trop Dis. 2020 Jan 21;14(1):e0007586. doi: 10.1371/journal.pntd.0007586 (PMC6994197; doi:10.1371/journal.pntd.0007586)
Supplement: S2 Table — Enrolled patients and the newborns were followed up post hospital discharge to ascertain their vital status. (DOCX) [file pntd.0007586.s003.docx]

| **Patient type** | **Timing of follow-up** | | |
| --- | --- | --- | --- |
|  | **1 week after delivery date** | **3 months after delivery date** | **3 months after hospital discharge** |
| Non-pregnant women and adult men |  |  | √ |
| Pregnant women delivered in hospital | √ | √ |  |
| Pregnant women discharged from hospital before 3rd trimester | √ | √ | √ |
| Pregnant women discharged from hospital during 3rd trimester | √ | √ |  |
