# Supplementary material for: Hepatitis E as a cause of adult hospitalization in Bangladesh: Results from an acute jaundice surveillance study in six tertiary hospitals, 2014-2017
Source: PLoS Negl Trop Dis. 2020 Jan 21;14(1):e0007586. doi: 10.1371/journal.pntd.0007586 (PMC6994197; doi:10.1371/journal.pntd.0007586)
Supplement: S3 Table — (DOCX) [file pntd.0007586.s004.docx]

| Characteristics | Rural (N=1494) | | Urban (N=431) | | Total (N=1925) | |
| --- | --- | --- | --- | --- | --- | --- |
|  | Patient tested | Anti-HEV IgM Positive | Patient tested | Anti-HEV IgM Positive | Patient tested | Anti-HEV IgM Positive |
|  |  | n (%) |  | n (%) |  | n (%) |
| Education |  |  |  |  |  |  |
| None | 316 | 51 (16) | 67 | 12 (18) | 383 | 63 (16) |
| Class 1-5 | 482 | 137 (28) | 92 | 38 (41) | 574 | 175 (31) |
| Class 6-11 | 460 | 175 (38) | 184 | 84 (46) | 644 | 259 (40) |
| Class 12 or more | 236 | 120 (51) | 88 | 44 (50) | 324 | 164 (51) |
| Monthly household expenditure in Bangladeshi taka |  |  |  |  |  |  |
| < 5000 (US$ 62) | 236 | 40 (17) | 34 | 8 (24) | 270 | 48 (18) |
| 5000-9,999 (US$ 63-125) | 595 | 171 (29) | 122 | 43 (35) | 717 | 214 (30) |
| 10,000-14,999 (US$ 126-187) | 314 | 110 (35) | 85 | 32 (38) | 399 | 142 (36) |
| ≥ 15,000 (US$ 188) | 201 | 79 (39) | 127 | 59 (46) | 328 | 138 (42) |
