# Supplementary figures and images for: Hepatitis E as a cause of adult hospitalization in Bangladesh: Results from an acute jaundice surveillance study in six tertiary hospitals, 2014-2017
Source: PLoS Negl Trop Dis. 2020 Jan 21;14(1):e0007586. doi: 10.1371/journal.pntd.0007586 (PMC6994197; doi:10.1371/journal.pntd.0007586)

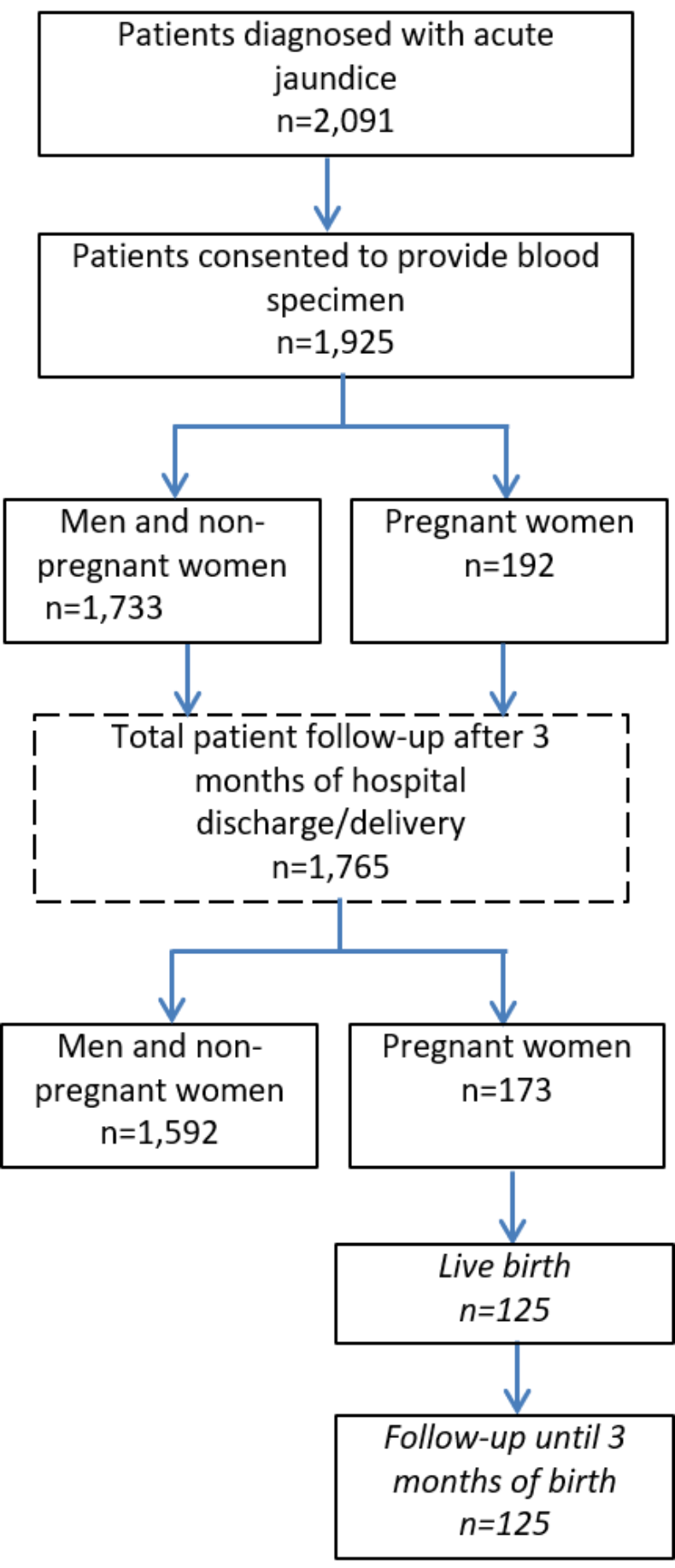

Supplement: S1 Fig — Number of patients diagnosed with acute jaundice, provided a blood specimen and followed up post hospital discharge in the six tertiary hospitals in Bangladesh. (TIF) [file pntd.0007586.s005.tif]
