# Supplementary material for: Hepatitis E as a cause of adult hospitalization in Bangladesh: Results from an acute jaundice surveillance study in six tertiary hospitals, 2014-2017
Source: PLoS Negl Trop Dis. 2020 Jan 21;14(1):e0007586. doi: 10.1371/journal.pntd.0007586 (PMC6994197; doi:10.1371/journal.pntd.0007586)
Supplement: Attachment — Submitted filename: Response to Reviewers comments_HEV surveillance.docx [file pntd.0007586.s006.docx]

Manuscript ID: PNTD-D-19-01051

**Hepatitis E as a cause of adult hospitalization in Bangladesh: Results from an acute jaundice surveillance study in six tertiary hospitals, 2014-2017**

**Response to reviewers’ comments:**

**Reviewer: 1**

**Comment:** The objectives were clear and the method appropriate. There was a robust sample size well distributed throughout the country to represent a national burden for severe hospitalized jaundice. As acknowledged by the authors, the design did not allow for estimates of milder disease, or those with severe disease but seeking traditional medicine.

►**Response**: We thank the reviewer for their comments and agree that this study estimates only the prevalence of HEV infection among severely ill patients with acute jaundice presenting to these tertiary care hospitals. While many patients with HEV will be missing from our study, our data comprise the most comprehensive data available on the geographic distribution of HEV transmission in Bangladesh.

**Comment:** The results are clearly represented. It seems that all causes of jaundice have a negative impact on pregnancies. Although there were more neonatal deaths with HEV + pregnant women, maternal mortality and miscarriage were equally bad among HEV+ and HEV- pregnant women. This might have been brought up in the discussion more fully. Hepatitis is bad for maternal and fetal outcomes regardless the type.

►**Response**: In response to this comment, we have now included the following paragraph in the discussion section:

*“Although males and non-pregnant females who were HEV IgM negative were more likely to die than those with HEV infection, the case fatality in pregnancy was similar between both groups. Furthermore, the occurrence of stillbirths and miscarriages was also common in both HEV and non-HEV acute jaundice patients. However, the neonatal mortality among children born to mothers with HEV infection was significantly higher than for children born to mothers without HEV infection. The higher proportion of neonatal deaths among mothers with HEV infection might be explained by the well-described risk of maternal to child transmission of HEV, or the frequency of pre-term delivery (Ref: Tsega E et al., 1992; Khuroo et al., 2003; Kamar A et al., 2004). However, further research is required to understand the contribution of the vertical transmission of HEV to fetal and neonatal mortality, and the mechanism of maternal response to HEV infection.”*

**Comment:** Conclusions: The conclusions are supported by the evidence and the limitations nicely acknowledged.

►**Response**: No response required.

**Comment:** This is an important paper which well describes the burden of HEV among severe jaundice cases seeking hospital care in Bangladesh. While the lack of quality burden of disease data is an important reason why there is not clear and strong guidance regarding use of the HEV vaccine, it is not the only limitation. There is insufficient experience with the vaccine in high risk populations , like pregnant women or those in humanitarian emergency settings or other areas (like Bangladesh) where genotype 1 and fecal-oral transmission is the common route. 
It is unfortunate that those under 14 yo and hospitalized with jaundice could not be included, or that families were not investigated to find additional mild or asymptomatic cases. But I understand the limitations of funding. Hopefully these important results will lead to additional studies to extend the understanding of HEV transmission into the household and to younger ages. If vaccines are to be used most effectively, the entire transmission cycle should be known.

►**Response**: We thank the reviewer for their comments.

**Reviewer: 2**

**Comment:** The objective of this study was to estimate the prevalence of HEV infection among jaundice patients aged >=14 and admitted in tertiary hospitals in Bangladesh, estimate the case-fatality rate and estimate factors associate with mortality, as well as describe seasonal and geographical trends
The description of objective is very complex. Which is the main objective of the study? Please rewrite this section and specify the main objective.

►**Response**: We have now clarified the objectives as follows:

*“The main objectives of this study were to estimate the prevalence of HEV infection among acute jaundice patients aged ≥14 years admitted to tertiary hospitals in Bangladesh and estimate the case-fatality among these patients. Secondary objectives included describing seasonal and geographic trends in the prevalence of HEV disease, identifying factors associated with mortality, measuring pregnancy outcomes, and neonatal mortality of children born to mothers with acute HEV infection.”*

**Comment:** The study is well designed, but please insert the list of items included in the case report (line 138) and a schema of the method of enrollement of patients

►**Response**: We have now included a list of clinical signs and symptoms of acute jaundice patients that were collected at the time of hospital admission as an appendix (S1 Table). We have mentioned this appendix in the Method section (at the end of 2nd paragraph of Method Section, Page 9) and in the Result section (texts related to Table 2, Page 17). A flow chart of patient enrolment is an existing appendix (S1 Fig).

**S1 Table: List of clinical signs and symptoms of acute jaundice patients collected at the time of admission in the surveillance hospitals (N=1925)**

| Symptom | Is symptom present at any time during current illness?  (Yes/No)  n (%) | How many days ago did patient first experience symptom?  (days)  Median (IQR) | Is symptom continuing on the day of hospital admission?  (Yes/No)  n (%) |
| --- | --- | --- | --- |
| Fever | 1717 (89%) | 15 (8-30) | 794 (41%)) |
| Yellow skin | 1918 (100%) | 12 (7-21) | 1901 (99%) |
| Yellow eyes | 1922 (100%) | 14 (7-25) | 1916 (100%) |
| Dark color urine | 1910 (99%) | 15 (5-30) | 1892 (98%) |
| Nausea | 1602 (83%) | 12 (7-21) | 898 (47%) |
| Vomiting | 1304 (68%) | 8 (5-15) | 398 (21%) |
| Loss of appetite | 1556 (81%) | 15 (9-30) | 1407 (73%) |
| Abdominal pain | 1175 (61%) | 12 (7-28) | 886 (43%) |
| Haematemesis | 74 (4%) | 5 (3-10) | 16 (1%) |
| Diarrhoea | 327 (17%) | 7 (4-15) | 77 (4%) |
| Constipation | 826 (43%) | 10 (7-20) | 492 (26%) |
| Melaena | 367 (19%) | 7 (4-15) | 123 (6%) |
| Headache | 694 (36%) | 10 (7-20) | 433 (23%) |
| Convulsion | 161 (8%) | 5 (2-10) | 43 (2%) |
| Unconsciousness | 152 (8%) | 3 (2-6) | 62 (3%) |
| Drowsiness | 776 (40%) | 15 (7-30) | 700 (36%) |
| Altered mental status | 146 (8%)) | 10 (4-30) | 109 (6%) |

**Comment:** The sample size was not reported in the method section, please add

►**Response:** This was a population-based surveillance project, so our goal was to identify all patients meeting the case definition and describe the prevalence and outcomes. Since we were not aiming to sample from a population, we had no sample size calculation.

**Comment:** The discussion and conclusion were based on univariate analysis. Please add multivariate analysis to validate results or univariate analysis

►**Response**: We assume that this suggestion is related to identification of risk factors for mortality among patients (Table 3). We have not pursued a multivariable model because our study was not powered to perform a multivariable analysis. In multivariable analysis, a rule of thumb is that only one predictor can be studied for every 10 events (Ref: Harrell, F. E. Jr et al, 1996; Peduzzi, Peter et al., 1996). In our study, we had only 28 HEV deaths, so multivariable analysis was not possible.

**Comment:** Please add number of ethic authorisation

►**Response**: In the section on ‘Human subjects’, we have now included the study protocol number, which is a unique identifier for each study approved by the icddr,b Ethical Review Committee.

**Comment**: As mentioned in method section authors have to add a multivariate analysis in order to validate results of the univariate analysis

►**Response**: Please see our response in the Method section above.

**Comment:** Please add median age and Min and Max in Table 1

►**Response**: We have now included median age and min-max in Table 1.

**Comment:** In Figure 2 authors declared that the proportions of patients anti-HEV IgM declined significantly with time after two months but IC95% from the week 2 to four overlap. Please modify this sentence.

►**Response**: In the revised version of manuscript, we have modified Figure 2. The modification was required as we reconsidered the definition of date of onset of illness. Previously, we defined the onset of illness as the date when the patient first reported experiencing yellow eyes or skin or nausea/vomiting. But in the revised version, we defined the onset of illness as the date when any sign and symptom developed, including experiencing yellow eyes or skin or nausea/vomiting, or also fever, anorexia, abdominal pain, melaena, or unconsciousness (Data analysis part of Methods Section).

We have also revised the text related to Figure 2 in the Result Section.

**Revised wording and Figure:** *“Patients who were admitted to hospital between three and four weeks of onset of illness had the highest anti-HEV IgM positivity (46%; Fig 2).* *The proportion who were anti-HEV IgM positive declined with time after four weeks of onset of illness (χ2 for trend: 8.1; p <0.01).”*

**Previous wording and Figure**: “Patients who were admitted to hospital between one and two weeks of onset of illness had the highest anti-HEV IgM positivity (42%; Fig 2). The proportion who were anti-HEV IgM positive declined with time after two weeks of onset of illness (χ2 for trend: 21.3; *p <0.001)*.”

**Comment:** Figure 3: How do you explain the increase of the number of IgM positive cases during the 2016 summer?

►**Response**: To investigate the increase, we have revised Figure 2 to present the number of acute jaundice cases by month for each hospital as a stacked graph. As a result of this investigation, we identified increased numbers in 2 hospitals. To highlight this, we have added the following lines in the Discussion section of revised manuscript:

*“……….However, an increase in the number of HEV IgM positive cases was reported from the Chittagong Medical College Hospital during Sep-Oct, 2016 and from the Barishal Medical College Hospital during Jun-Sep, 2017. The unusually high number of cases suggests that there might have been an outbreak in those months which was undetected in real-time, perhaps due to delays in laboratory testing. ……”*

Below is the revised Figure 2:

No. of HEV IgM positive cases

| Month of onset of jaundice | Bogra | Barishal | Kishoregonj | Chittagong | Sylhet | Dhaka |
| --- | --- | --- | --- | --- | --- | --- |
| 2015 Apr | 0 | 4 | 0 | 1 | 1 | 4 |
| 2015 May | 2 | 0 | 1 | 1 | 1 | 6 |
| 2015 Jun | 3 | 1 | 2 | 0 | 2 | 3 |
| 2015 Jul | 0 | 4 | 2 | 1 | 1 | 3 |
| 2015 Aug | 0 | 8 | 2 | 3 | 1 | 5 |
| 2015 Sep | 0 | 5 | 0 | 7 | 0 | 5 |
| 2015 Oct | 0 | 3 | 1 | 6 | 0 | 4 |
| 2015 Nov | 3 | 3 | 1 | 6 | 1 | 5 |
| 2015 Dec | 1 | 9 | 2 | 4 | 1 | 4 |
| 2016 Jan | 1 | 7 | 0 | 2 | 0 | 6 |
| 2016 Feb | 3 | 12 | 3 | 2 | 0 | 6 |
| 2016 Mar | 2 | 5 | 0 | 2 | 1 | 3 |
| 2016 Apr | 1 | 5 | 1 | 5 | 1 | 8 |
| 2016 May | 2 | 6 | 0 | 2 | 1 | 3 |
| 2016 Jun | 1 | 5 | 0 | 1 | 0 | 1 |
| 2016 Jul | 4 | 5 | 1 | 9 | 0 | 5 |
| 2016 Aug | 4 | 4 | 2 | 6 | 2 | 7 |
| 2016 Sep | 1 | 6 | 0 | 18 | 2 | 8 |
| 2016 Oct | 7 | 6 | 1 | 14 | 1 | 6 |
| 2016 Nov | 1 | 4 | 0 | 11 | 0 | 5 |
| 2016 Dec | 0 | 1 | 2 | 9 | 2 | 5 |
| 2017 Jan | 0 | 3 | 3 | 7 | 1 | 8 |
| 2017 Feb | 3 | 4 | 1 | 9 | 0 | 8 |
| 2017 Mar | 9 | 5 | 0 | 7 | 4 | 7 |
| 2017 Apr | 3 | 4 | 4 | 2 | 5 | 5 |
| 2017 May | 8 | 4 | 1 | 6 | 2 | 13 |
| 2017 Jun | 3 | 11 | 0 | 5 | 3 | 7 |
| 2017 Jul | 6 | 11 | 1 | 4 | 1 | 8 |
| 2017 Aug | 4 | 14 | 0 | 5 | 0 | 3 |
| 2017 Sep | 0 | 14 | 0 | 2 | 3 | 1 |

Below is the Figure 2 in the previous version:

**Comment:** Line 304-305 please place after line 301

No. of IgM positive cases

| Month of onset of illness | Anti-HEV IgM positive patients |
| --- | --- |
| 2015 Apr | 10 |
| 2015 May | 11 |
| 2015 Jun | 11 |
| 2015 Jul | 11 |
| 2015 Aug | 19 |
| 2015 Sep | 17 |
| 2015 Oct | 14 |
| 2015 Nov | 19 |
| 2015 Dec | 21 |
| 2016 Jan | 16 |
| 2016 Feb | 26 |
| 2016 Mar | 13 |
| 2016 Apr | 21 |
| 2016 May | 14 |
| 2016 Jun | 8 |
| 2016 Jul | 24 |
| 2016 Aug | 25 |
| 2016 Sep | 35 |
| 2016 Oct | 35 |
| 2016 Nov | 21 |
| 2016 Dec | 19 |
| 2017 Jan | 22 |
| 2017 Feb | 25 |
| 2017 Mar | 32 |
| 2017 Apr | 23 |
| 2017 May | 34 |
| 2017 Jun | 29 |
| 2017 Jul | 31 |
| 2017 Aug | 26 |
| 2017 Sep | 20 |

►**Response**: We have moved the line as suggested. Please see below the updated paragraph:

*“Among the 192 enrolled pregnant women, we followed up 173 (90%) until the outcome of their pregnancy. Among the 66 mothers who were anti-HEV IgM positive, 53 (80%) had a live birth and among the 107 mothers who were anti-HEV IgM negative, 72 (67%) had a live birth (Table 4). Nineteen percent (10/53) of the live-born babies born to mothers with acute HEV infection died compared to 7% (5/72) of babies born to women without HEV infection (p=0.038). The median age at death of the babies who were born to HEV infected mothers was two days (IQR: 1-3 days; all within one week) and was five days (IQR: 2-8 days) for those were born to HEV uninfected mothers (p=0.207). Of the 62 HEV infected mothers who were alive until the delivery, 9 (15%) had a miscarriage or stillbirth.”*

**Comment:** Table 4: pvalue of live birth/still birth are missed?

►**Response**: We have now added the p-value comparing the live births/still births among the Anti-HEV IgM positive and Anti-HEV IgM negative cases. Please see updated Table 4.

**Comment:** The authors need to improve statistical analysis

►**Response**: As per the reviewer’s suggestion, we have now included p-values in Table 4. The reviewer also suggested performing a multivariable analysis, however, we do not have a sufficient number of events to run a multivariable model. We have explained this in the response to a previous comment.
